# Primary obstructive megaureter

**DOI:** 10.11604/pamj.2020.37.296.26867

**Published:** 2020-12-02

**Authors:** Ahmed Ibrahimi, Idriss Ziani

**Affiliations:** 1 Department of Urology A, Faculty of Medicine and Pharmacy, Ibn Sina University Hospital, Mohammed V University, Rabat, Morocco

**Keywords:** Primary obstructive megaureter, hydroureteronephrosis, ureterovesical junction obstruction, ureteral reimplantation

## Image in medicine

Primary obstructive megaureter (POM) is an uncommon disease in adults, it is defined as an intrinsic congenital abnormality at the lower segment of the ureter in the ureterovesical junction. It is generally unilateral, but may be seen bilaterally in 15% to 25% of cases and occurs more often in males. Symptoms includes flank pain, recurrent urinary tract infection, hematuria and acute renal colic. Chronic ureteral obstruction may lead to serious complications such as infection, stone formation and renal failure. Primary symptomatic or complicated obstructive megaureter requires surgical management, using different surgical techniques, consisting of excision of the adynamic ureteral segment and reimplantation into the bladder. We present a case of 19-year-old young male, without past medical history, presented to the emergency department with intermittent recurrent left flank pain for the last three years, without lower urinary tract symptoms (LUTS), fever or haematuria. On clinical examination, his abdomen was soft to palpate, with no tenderness. Routine laboratory tests were normal. Urinary ultrasonography (US) revealed a giant left hydroureteronephrosis, with reduction in cortical thickness. Abdominal and pelvic computed tomography (CT) showed a left giant hydroureteronephrosis (A, B and C) and huge dilatation of left ureter with tortuosity down to the urinary bladder (B and C). These findings allowed us to retain the diagnosis of primary obstructed megaureter. The patient underwent an excision of the terminal narrow adynamic ureteral segment, with laparoscopic transperitoneal ureteral reimplantation according to the Lich-Gregoir technique. The postoperative course was completely uneventful. The patient was discharged from the hospital at the 5^th^ postoperative day without any complication. At the follow-up, he was healthy, with normal renal function, without dilatation of the collecting system or vesicoureteral reflux.

**Figure 1:**
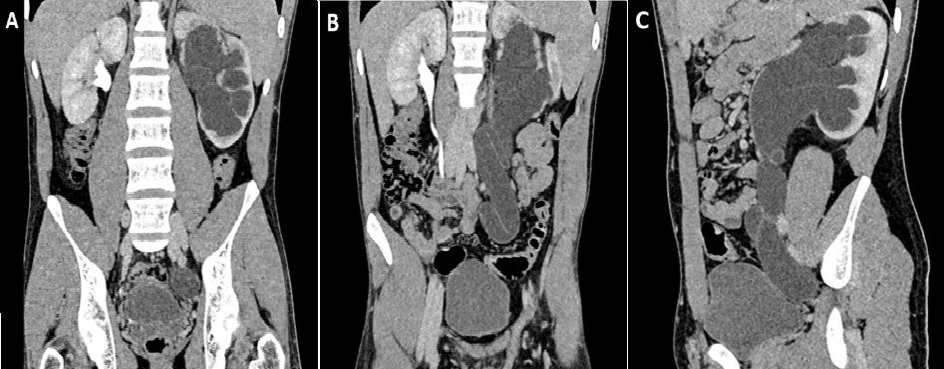
left primary obstructive megaureter: A) coronal reconstruction of the CT scan showing a left giant hydronephrosis, with marked reduction of the cortical thickness and dilation of the terminal segment of the ureter just before the ureterovesical junction; B) coronal reconstruction of the CT scan showing a left giant hydroureteronephrosis, with the absence of urinary excretion of contrast agent and huge dilatation of left ureter with tortuosity down to the urinary bladder; C) sagittal reconstruction of the CT scan showing an enlarged left kidney, with giant pelvis and ureter just before the ureterovesical junction

